# Bezafibrate improves postprandial hypertriglyceridemia and associated endothelial dysfunction in patients with metabolic syndrome: a randomized crossover study

**DOI:** 10.1186/1475-2840-13-71

**Published:** 2014-04-05

**Authors:** Yuko Ohno, Toru Miyoshi, Yoko Noda, Hiroki Oe, Norihisa Toh, Kazufumi Nakamura, Kunihisa Kohno, Hiroshi Morita, Hiroshi Ito

**Affiliations:** 1 Department of Cardiovascular Medicine, Okayama University Graduate School of Medicine, Dentistry and Pharmaceutical Sciences, Okayama, Japan; 2 Department of Cardiovascular Therapeutics, Okayama University Graduate School of Medicine, Dentistry and Pharmaceutical Sciences, 2-5-1 Shikata-cho, Okayama 700-8558, Japan; 3 Center of Ultrasonic Diagnostics, Okayama University Hospital, Okayama, Japan

**Keywords:** Atherosclerosis, Bezafibrate, Triglyceride, Endothelium, Vasodilation

## Abstract

**Background:**

Postprandial elevation of triglyceride-rich lipoproteins impairs endothelial function, which can initiate atherosclerosis. We investigated the effects of bezafibrate on postprandial endothelial dysfunction and lipid profiles in patients with metabolic syndrome.

**Methods:**

Ten patients with metabolic syndrome were treated with 400 mg/day bezafibrate or untreated for 4 weeks in a randomized crossover study. Brachial artery flow-mediated dilation (FMD) and lipid profiles were assessed during fasting and after consumption of a standardized snack. Serum triglyceride and cholesterol contents of lipoprotein fractions were analyzed by high-performance liquid chromatography.

**Results:**

Postprandial FMD decreased significantly and reached its lowest value 4 h after the cookie test in both the bezafibrate and control groups, but the relative change in FMD from baseline to minimum in the bezafibrate group was significantly smaller than that in the control group (-29.0 ± 5.9 vs. -42.9 ± 6.2 %, p = 0.04). Bezafibrate significantly suppressed postprandial elevation of triglyceride (incremental area under the curve (AUC): 544 ± 65 vs. 1158 ± 283 mg h/dl, p = 0.02) and remnant lipoprotein cholesterol (incremental AUC: 27.9 ± 3.5 vs. 72.3 ± 14.1 mg h/dl, p < 0.01). High-performance liquid chromatography analysis revealed that postprandial triglyceride content of the chylomicron and very low-density lipoprotein fractions was significantly lower in the bezafibrate group than in the control group (p < 0.05).

**Conclusion:**

Bezafibrate significantly decreased postprandial endothelial dysfunction, and elevations of both exogenous and endogenous triglycerides in patients with metabolic syndrome, suggesting that bezafibrate may have vascular protective effects in these patients.

**Clinical trial registration:**

Unique Identifiers: UMIN000012557

## Background

Non-fasting postprandial serum triglyceride (TG) concentrations have been shown to predict the risk of cardiovascular pathology more accurately than fasting TG concentrations, and this relationship is independent of traditional risk factors for coronary artery disease.[1,2] TG-rich lipoproteins (TRLs), which include chylomicrons (CMs) assembled from TGs, dietary cholesterol, and apolipoprotein B-48 (ApoB-48), are highly atherogenic, [3,4] because postprandial TRL elevation occurs in conjunction with the production of proinflammatory cytokines and oxidative stress, resulting in endothelial dysfunction [5,6]. Therefore, identification of novel therapeutic approaches to lower postprandial concentrations of serum lipids is of great clinical interest.

Fibrates are one of the most important classes of medications currently used to treat atherogenic dyslipidemia [7]. Bezafibrate is the only clinically available peroxisome proliferator-activated receptor agonist that acts on all three receptor subtypes (α, β, and δ) [8]. Bezafibrate improves serum lipid profiles [8] insulin sensitivity 2 [9] and fasting endothelial function [10,11]. Several studies have shown favorable effects of fibrates on postprandial TG elevation, but the reported effects on postprandial endothelial dysfunction are ambiguous. Improved flow-mediated dilation (FMD) after oral fat loading has been shown in patients with type 2 diabetes mellitus after 12 weeks of ciprofibrate therapy, [12] but no such benefits were observed in healthy volunteers after 3 weeks of gemfibrozil therapy [13]. Only one study has examined the effect of bezafibrate directly on postprandial lipemia [14]. However, its effects on postprandial endothelial dysfunction have not been investigated. The aim of this study was to investigate the effects of bezafibrate on postprandial TRLs and postprandial lipemia-induced endothelial dysfunction in patients with metabolic syndrome.

## Methods

This study was approved by the Ethics Committee of Okayama University Graduate School of Medicine, Dentistry, and Pharmaceutical Sciences, and written informed consent was obtained from all participants before beginning the protocol. This study was conducted according to the principles expressed in the Declaration of Helsinki and is registered in the UMIN Clinical Trials Registry (UMIN000012557).

### Participants and design

**Figure 1:**
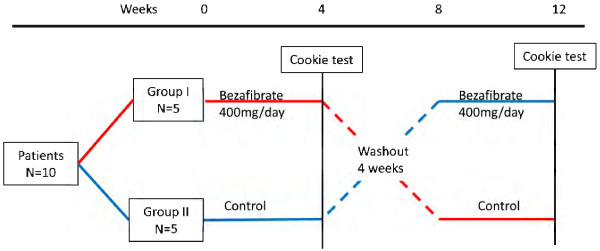
Study design.

This was a randomized crossover study and all procedures were performed at Okayama University Hospital. After the possible consequences of the study were explained, all participants provided written informed consent prior to screening and study enrollment. Eligible subjects were patients diagnosed with metabolic syndrome ranging in age from 20 to 85 years. All participants underwent pre-study medical exams and medical history interviews. Metabolic syndrome was diagnosed using the definition of the International Diabetes Federation [15]. In male Asians, central obesity is required (≥80 cm) for a diagnosis of metabolic syndrome in addition to at least two of the following: blood pressure of ≥130/85 mmHg and/or current use of antihypertensive medication; fasting plasma glucose of ≥100 m/dl and/or current use of antidiabetic medication; TG of ≥150 mg/dl; and high-density lipoprotein cholesterol (HDL-C) of <40 mg/dl. The study consisted of two 4-week crossover treatment periods in which half of the participants (chosen randomly) were administered 400 mg/day bezafibrate orally and the other half of the participants did not receive bezafibrate. In the second 4-week period, the groups were reversed (Figure 1). A “cookie test”, where subjects received a standardized snack, was performed after the 4-week treatment period. There was a 4-week period between the two phases during which neither group received bezafibrate. The pre-specified primary outcome measure was the difference in the decrease in FMD after the cookie test between the bezafibrate and control groups, and the secondary outcome measure was the difference in lipid profiles after the cookie test between the bezafibrate and control groups.

### Cookie test protocol

After fasting overnight for at least 8 h, a cookie test was performed [16]. The cookie consisted of 75 g carbohydrate, 28.5 g fat, and 8 g protein for a total of 592 kcal per cookie (Saraya Corp., Osaka, Japan) [17]. Subjects were given the amount of cookies equivalent to 30 g fat/m^2^ body surface area and were instructed to ingest the cookies with water within 20 min. The experimental starting point began when half of the cookies had been ingested. Venous blood samples were drawn and FMD was measured during the fasting state before and 2, 4, 6, and 8 h after the cookie test. The participants were instructed not to eat anything else for 8 h after eating the cookies. FMD was measured by a single technician who was blinded to the study design and treatment status of the participants.

### Measurement of biochemical parameters

The following parameters were measured in the fasting state before cookie ingestion: serum total cholesterol (Total-C), TG, low-density lipoprotein cholesterol (LDL-C), HDL-C, remnant lipoprotein cholesterol (RLP-C), ApoB-48, pentraxin 3, plasma insulin, plasma glucose, and hemoglobin A1c. These measurements were performed by SRL Company, Ltd. (Tokyo, Japan). Homeostasis model assessment ratio (HOMA-R) was calculated as “Fasting glucose × Fasting insulin)/405”. Serum cholesterol and TG contents of lipoprotein fractions were analyzed by high-performance liquid chromatography performed by Skylight Biotech (Akita, Japan), as described previously [18]. Homeostasis model assessment of insulin resistance (HOMA-IR) was calculated as fasting plasma glucose (mg/dl) × fasting plasma insulin (μU/ml)/405. Serum total-C, TG, LDL-C, HDL-C, RLP-C, ApoB-48, plasma glucose, plasma insulin, and pentraxin 3 were measured 2, 4, 6, and 8 h after the cookie test. To compare the postprandial changes in these parameters before and after treatment for 4 weeks, AUC was calculated using the trapezoidal method.

### Measurement of FMD

FMD was measured according to the published guidelines for ultrasound assessment of FMD of the brachial artery [19]. Using a 10-MHz linear-array transducer probe (Unex Company Ltd., Nagoya, Japan), longitudinal images of the brachial artery at baseline were recorded with a stereotactic arm, and artery diameter was measured after supine rest for ≥5 min. The artery diameter was measured from clear anterior (media-adventitia) and posterior (intima-media) interfaces, which were determined manually. Suprasystolic compression (50 mmHg higher than systolic blood pressure) was performed at the right forearm for 5 min, and the artery diameter was measured continuously from 30 s before to ≥2 min after cuff release. All measurements of FMD were made by a single technician blinded to drug allocation; the intra- and inter-observer correlation coefficients were high (>0.9).

### Statistical analysis

Sample size was determined on the basis of the estimated FMD reported in a recent study [20]. From this estimate, we assumed a mean improvement in postprandial % FMD of 2.7% with standard deviation of 2.0% by bezafibrate administration. To use a two-sided test for differences between groups, a minimal sample size of 8 participants was required to detect statistical differences in % FMD with a power of 90% and an α-type error of 5%. Results and data in the figures are expressed as mean ± standard error (SE). Differences in lipid profiles and endothelial function between the two groups were compared using the Wilcoxon signed -rank test. Spearman correlation coefficients were used to assess relationships between maximum reduction in % FMD and lipid and glucose profiles. Values of p < 0.05 were considered significant.

## Results

### Characteristics of participants during fasting

The mean age of the participants was 43 ± 10 years, and the mean body mass index was 28.8 ± 1.2 kg/m^2^. All participants were men. Table 1 shows the lipid/lipoprotein profiles and the glycemic parameters of the participants following each 4-week phase of control or treatment with 400 mg/day bezafibrate. Bezafibrate treatment significantly decreased the fasting levels of total-C, TG, RLP-C, and ApoB-48 but did not affect the levels of LDL-C, HDL-C, plasma glucose, insulin, hemoglobin A1c, and pentraxin 3. No significant differences were observed in systolic and diastolic blood pressure following bezafibrate treatment.

**Table 1:** Characteristics of fasted participants after a 4-week administration of bezafibrate or control

	**Bezafibrate**	**Control**	**p**
Body mass index (kg/m^2^)	28.8 ± 1.2	28.6 ± 1.1	0.24
Systolic blood pressure (mmHg)	127 ± 4	125 ± 4	0.34
Diastolic blood pressure (mmHg)	78 ± 3	80 ± 2	0.98
Heart rate (beats/min)	64 ± 2	65 ± 1	0.20
**Laboratory data**			
Total-C (mg/dl)	217 ± 9	197 ± 8	0.02
LDL-C (mg/dl)	129 ± 6	135 ± 9	0.57
HDL-C (mg/dl)	46 ± 3	43 ± 3	0.22
TG (mg/dl)	135 ± 24	203 ± 35	<0.01
RLP-C (mg/dl)	5.7 ± 0.7	7.9 ± 1.3	0.02
ApoB-48 (μg/ml)	3.5 ± 0.7	6.0 ± 1.5	0.02
Glucose (mg/dl)	97 ± 3	101 ± 5	0.15
Insulin (μU/ml)	12.7 ± 4.8	8.5 ± 1.4	0.43
HOMA-IR	2.9 ± 0.2	2.1 ± 0.2	0.48
Hemoglobin A1c (%)	5.2 ± 0.3	5.3 ± 0.3	0.17
Pentraxin 3 (ng/ml)	1.5 ± 0.2	1.5 ± 0.2	0.64
**Endothelial function**			
Brachial artery diameter (mm)	4.3 ± 0.2	4.3 ± 0.2	0.70
% FMD	6.9 ± 0.7	5.9 ± 0.7	0.04

Data are mean ± SE. Total-C, total cholesterol; LDL-C, low-density lipoprotein cholesterol; HDL-C, high-density lipoprotein cholesterol; TGs, triglycerides; RLP-C, remnant lipoprotein cholesterol; ApoB-48, apolipoprotein B-48; HOMA-IR, homeostasis model assessment of insulin resistance; FMD, flow-mediated dilation.

### Postprandial endothelial function

**Figure 2:**
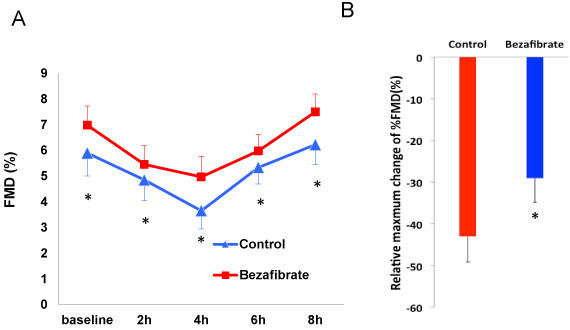
**Postprandial changes in % FMD after the cookie test in the control and bezafibrate groups (A) and the relative maximum change in % FMD (B).** Data are expressed as mean ± SE. *p < 0.05 vs. control group.

Figure 2 presents the comparison of postprandial endothelial function, which was assessed by % FMD, between the bezafibrate and control groups. In both groups, postprandial % FMD decreased significantly after the cookie test and reached its lowest value at 4 h. The decrease in postprandial FMD relative to the baseline was significantly improved by bezafibrate treatment but not by the control (-29.0 ± 5.9 vs. -42.9 ± 6.2%, p = 0.04).

### Postprandial lipid and glucose parameters

**Figure 3:**
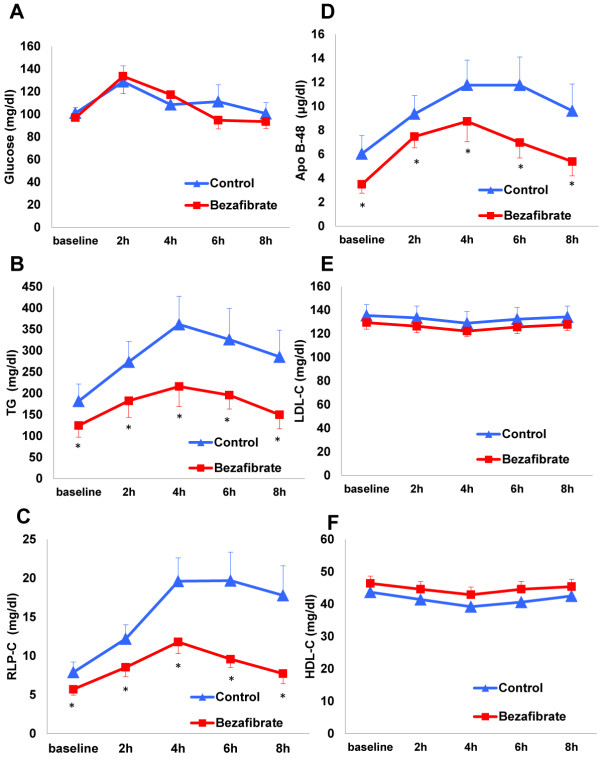
**Serial postprandial changes in glucose (A), TG (B), RLP-C (C), ApoB-48 (D), LDL-C (E), and HDL-C (F) in the control and bezafibrate groups.** Data are expressed as mean ± SE. *p < 0.05 vs. control group.

Serial changes in the levels of lipids/lipoproteins, glucose, insulin, and pentraxin 3 in postprandial samples following each 4-week phase of control or bezafibrate treatment are shown in Figure 3 and Additional file 1: Table S1. In the control group, the postprandial levels of serum TG, RLP-C, and ApoB-48 increased over time, peaked at 4 h, and then decreased, and the levels of glucose and insulin increased, peaked at 2 h, and then decreased. The levels of total-C, LDL-C, HDL-C, and pentraxin 3 did not change significantly during the postprandial period in the control group.

The changes in postprandial TGs, RLP-C, and ApoB-48 at 4 h were significantly smaller in the bezafibrate group than in the control group (Figure 3). The incremental AUCs for serum TG and RLP-C were significantly smaller in the bezafibrate group than in the control group (TG: 544 ± 65 vs. 1158 ± 283 mg h/dl, p = 0.02; RLP-C: 27.9 ± 3.5 vs. 72.3 ± 14.1 mg h/dl, p < 0.01), whereas no differences in the incremental AUC for ApoB-48 were observed (27.3 ± 4.2 vs. 33.2 ± 5.1 μg h/ml, p = 0.31). The total AUC for total-C in the bezafibrate group was significantly smaller than that in the control group (1580 ± 72 vs. 1749 ± 76 mg h/dl), No differences in the total AUC for LDL-C and HDL-C were observed between the bezafibrate and control groups (LDL-C: 1005 ± 41 vs. 1058 ± 76 mg h/dl, p = 0.52; HDL-C: 356 ± 18 vs. 328 ± 10 mg h/dl, p = 0.14). Moreover, no significant differences in the total AUC for glucose, insulin, or pentraxin 3 were observed between the bezafibrate and control groups (glucose: 881 ± 75 vs. 898 ± 89 mg h/dl, p = 0.52; insulin: 344 ± 83 vs. 266 ± 51, p = 0.29; pentraxin 3: 11.0 ± 1.0 vs. 11.1 ± 0.9 ng h/ml, p = 0.87).

The relationships of FMD with lipid and glucose parameters at baseline and postprandially, and their corresponding changes in response to the cookie test in the bezafibrate and control groups, were evaluated (Tables 2 and 3). The associations of FMD with TRLs and glucose parameters at baseline were mild to moderate. Change in FMD showed mild to moderate correlations with changes in postprandial TG, RLP-C, and ApoB-48 in either group. Change in FMD showed almost no correlations with changes in glucose, insulin, and HOMA-R.

**Table 2:** Correlations between FMD and lipid and glucose parameters at baseline and postprandially and changes in these parameters in response to the cookie test in the control group

**Dependent variable, FMD**	**Baseline**		**4 h**		**Change**	
	**r**	**p**	**r**	**p**	**r**	**p**
TG	-0.42	0.23	-0.23	0.53	-0.51	0.13
RLP-C	-0.41	0.24	-0.36	0.31	-0.54	0.10
ApoB-48	-0.23	0.53	-0.19	0.59	-0.42	0.23
	**Baseline**		**2 h**		**Change**	
	**r**	**p**	**r**	**p**	**r**	**p**
Glucose	-0.33	0.36	-0.16	0.65	-0.07	0.86
Insulin	-0.31	0.39	-0.21	0.57	-0.13	0.71
HOMA-R	-0.36	0.31	-0.17	0.65	-0.08	0.91

FMD, flow-mediated dilation; TG, triglyceride; RLP-C, remnant lipoprotein cholesterol; ApoB-48, apolipoprotein B-48; HOMA-R, Homeostasis model assessment ratio.

**Table 3:** Correlations between FMD and lipid and glucose parameters at baseline and postprandially, and changes in these parameters in response to the cookie test in the bezafibrate group

**Dependent variable, FMD**	**Baseline**		**4 h**		**Change**	
	**r**	**p**	**r**	**p**	**r**	**p**
TG	-0.33	0.35	-0.21	-0.60	-0.41	0.24
RLP-C	-0.39	0.27	-0.15	0.68	-0.26	0.47
ApoB-48	-0.39	0.27	-0.29	0.42	-0.37	0.29
	**Baseline**		**2 h**		**Change**	
	**r**	**p**	**r**	**p**	**r**	**p**
Glucose	-0.47	0.17	-0.51	0.13	0.004	0.99
Insulin	-0.33	0.36	-0.41	0.24	0.14	0.7
HOMA-R	-0.38	0.27	-0.47	0.17	0.16	0.66

FMD, flow-mediated dilation; TG, triglyceride; RLP-C, remnant lipoprotein cholesterol; ApoB-48, apolipoprotein B-48; HOMA-R, Homeostasis model assessment ratio.

**Figure 4:**
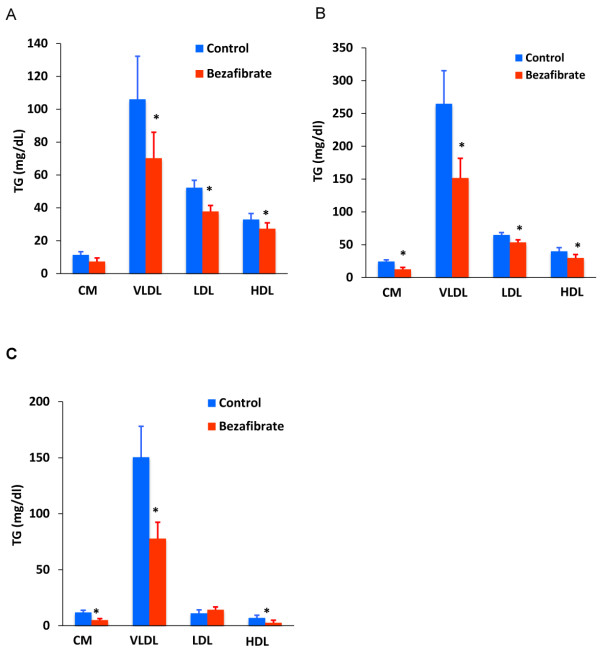
**TG content of lipoprotein fractions in the size range of CM, VLDL, LDL, and HDL in the control and bezafibrate groups in the fasting state (A) and 4 h after cookie ingestion (B).** The change in the TG content of lipoprotein fractions in the size range of CM, VLDL, LDL, and HDL from the fasting state to 4 h after cookie ingestion **(C)**. Data are expressed as mean ± SE. *p < 0.05 vs. control group.

To further examine postprandial lipid changes, cholesterol and TG concentrations in lipoprotein fractions in the size range of CM, very low-density lipoprotein (VLDL), LDL, and HDL were compared between the bezafibrate and control groups (Figure 4 and Additional file 2: Table S2). The fasting TG content of the VLDL, LDL, and HDL fractions was significantly reduced after bezafibrate treatment (Figure 4A). The postprandial TG content (4 h after the cookie test) of the CM, VLDL, LDL, and HDL fractions was also reduced in the bezafibrate group compared with the control group (Figure 4B). Furthermore, the change in TG content from fasting to 4 h was significantly reduced in the CM, VLDL, and HDL fractions in the bezafibrate group compared with the control group (Figure 4C). Fasting cholesterol concentrations in the LDL fractions and postprandial cholesterol concentrations in the CM and VLDL fractions were slightly, but significantly, lower in the bezafibrate group than in the control group (Additional file 2: Table S2).

## Discussion

This study demonstrated that bezafibrate significantly improved postprandial endothelial dysfunction and reduced both exogenous and endogenous postprandial TG levels in patients with metabolic syndrome. The potential association between the improvement in endothelial dysfunction and the decrease in TRLs suggests that bezafibrate may have vascular protective effects. Consequently, the risk of cardiovascular disease, especially the risk associated with postprandial TGs, may be reduced with bezafibrate treatment.

Our results are consistent with previous studies demonstrating reduced postprandial TG in response to fibrate treatment [12,21]. Evans et al. reported that ciprofibrate treatment for 3 months improved postprandial endothelial dysfunction and postprandial TG levels after ingestion of a test meal containing 80 g fat in patients with type 2 diabetes mellitus [12]. Rosenson et al. reported that fenofibrate treatment for 6 weeks significantly ameliorated postprandial hypertriglyceridemia, oxidative stress and inflammatory response after ingestion of a test meal containing standardized fat (50 g/m^2^) in patients with hypertriglyceridemia and the metabolic syndrome [21]. Blood TG concentrations reflect the balance between uptake of TG into and clearance from the circulation. The majority of TG secreted into the bloodstream after a high-fat meal is from fat absorption via enterocytes [22]. We demonstrated that intestinal TGs in chylomicrons and TG in VLDLs are decreased by bezafibrate. This is consistent with the findings of an experimental study, which found that postprandial chylomicrons and VLDLs were reduced in the plasma of bezafibrate-treated mice [23]. CM and VLDL-sized particles have been reported to include chylomicron remnants, suggesting that the postprandial TG-lowering effects of bezafibrate are mainly due to decreased uptake of TG into the circulation and increased clearance of TGs from the bloodstream [24]. However, a formal kinetic approach is required to confirm these findings and to evaluate the precise underlying mechanisms.

In this study, the changes in TRLs were moderately correlated with changes in FMD, although these correlations did not reach statistical significance. The sample size (n = 10) may have been too small to detect statistical significance, or bezafibrate may have a direct favorable effect on vascular function. Alternatively, improvements in vasodilation following fibrate therapy may have been due to changes in lipoprotein profiles. Indeed, bezafibrate can protect endothelial function in various populations including patients with metabolic syndrome [11] or coronary artery disease, [10] and we found that fasting % FMD was significantly improved in the bezafibrate group compared with the control group. Furthermore, bezafibrate increases the expression of endothelial nitric oxide synthase in cultured endothelial cells and increases nitric oxide bioactivity [25,26]. Suppression of systemic inflammation by peroxisome proliferator-activated receptor α activation is an additional mechanism whereby bezafibrate may enhance nitric oxide synthase activity [27]. Thus, bezafibrate treatment for 4 weeks may have preventive effects against postprandial endothelial dysfunction. Postprandial TRL-induced inflammation and oxidative stress, which affect the metabolism of nitric oxide and the release of vasoconstrictive mediators, result in endothelial dysfunction [6,28]. In our study, we evaluated the levels of pentraxin 3 as a marker of inflammation. Pentraxin 3 is produced by vascular endothelial cells and may more directly reflect the inflammatory status of the vasculature [29-31]. Therefore, an increase in pentraxin 3 after the cookie test was expected, but no significant changes were observed both in the control and bezafibrate groups. Therefore, further studies are necessary to determine whether the administration of bezafibrate limits postprandial inflammation and oxidative stress and to evaluate the interplay between these stresses and endothelial function.

Our finding showed that the baseline triglyceride concentration was much lower in the bezafibrate than in the control group and that baseline TG was significantly correlated with baseline ApoB-48 concentration (r > 0.9, p < 0.01, data not shown). Baseline ApoB-48 may affect the peak TG concentration after the cookie test, because the assembly of CM in enterocytes depends on baseline apoB-48 concentration. Thus, baseline TG may represent the postprandial response.

Previous large studies reported that bezafibrate increases HDL-cholesterol concentrations by over 10% [32-35]. In this study, bezafibrate increased HDL-cholesterol concentration by 3 mg/dl (about 7 %), although this difference did not reach statistical significance. The four-week duration of bezafibrate treatment may have been too short to significantly increase HDL-cholesterol concentrations. Bezafibrate has been shown to benefit patients with atherogenic dyslipidemia. As our study population consisted of patients with the metabolic syndrome, it was not unexpected that the increase in HDL-cholesterol would be less than observed in patients with diabetes mellitus and atherogenic dyslipidemia. In contrast, a recent study showed that bezafibrate treatment of patients with type 2 diabetes mellitus increased cholesterol efflux, but had no effect on the anti-inflammatory activity of HDL [36]. Thus, bezafibrate may have specific effects on HDL concentrations and functions.

In clinical trials, bezafibrate has been highly effective at reducing cardiovascular disease risk in patients with metabolic syndrome or atherogenic dyslipidemia [32,37]. A prospective observational study found that bezafibrate significantly improved HbA1c in dyslipidemic patients with diabetes in Japan [35]. At present, statins are the most widely applied therapy for the treatment and prevention of cardiovascular diseases related to atherosclerosis [38,39]. Despite the increased use of statins as a monotherapy for elevated LDL-C, a significant residual risk of cardiovascular disease remains for patients with atherogenic dyslipidemia and insulin resistance, which are typical in patients with type 2 diabetes mellitus and metabolic syndrome. Combined bezafibrate–statin therapy is more effective for achieving comprehensive lipid control and reducing cardiovascular disease risk [8,37,40,41].

Our study has several limitations. First, this was a single-blind study, and the number of participants enrolled was small. Therefore, some selection bias may have occurred. Second, no widely accepted method has been established for assessing postprandial hyperlipemia. The oral cookie test—with a defined quantity of fat per body surface area—may be a reliable method for detecting postprandial metabolic disturbances. The oral cookie test used in this study contains a greater content of carbohydrates than fat loading meals. These high-carbohydrate meals have a greater effect on glycemic parameters, including glucose and insulin concentrations, than high-fat meals. These metabolic differences may affect postprandial endothelial function and the effects of bezafibrate. Finally, because patients were only treated with bezafibrate for 4 weeks, we were not able to evaluate the long-term effects of bezafibrate on postprandial lipid dynamics.

## Conclusions

In this crossover study, we demonstrated that bezafibrate was effective at reducing postprandial TRL elevation and the accompanying induction of postprandial endothelial dysfunction in patients with metabolic syndrome. Bezafibrate may be useful in reducing future cardiovascular disease by ameliorating postprandial endothelial dysfunction in these patients.

## Abbreviations

ApoB-48: Apolipoprotein B-48; AUC: Area under the curve; CM: Chylomicron; FMD: Flow-mediated dilation; HDL-C: High-density lipoprotein cholesterol; HOMA-IR: Homeostasis model assessment of insulin resistance; LDL-C: Low-density lipoprotein cholesterol; RLP-C: Remnant lipoprotein cholesterol; TG: Triglyceride; Total-C: Total cholesterol; TRLs: TG-rich lipoproteins; VLDL: Very low-density lipoprotein.

## Competing interest

The authors declare that they have no competing interests.

## Authors' contributions

YO, TM, YN, HO, and NT conceived the study, and participated in its design and coordination and helped to draft the manuscript.KN, KK, HM, HI were involved in drafting the manuscript or critically revising it. All authors read and approved the final manuscript.

## Supplementary Material

Additional file 1: Table S1 Postprandial changes in lipid profiles and glycemic parameters in the bezafibrate and control groups. Click here for file

Additional file 2: Table S2 TG and cholesterol concentrations in lipoprotein fractions in the chylomicron, VLDL, LDL, and HDL fractions. Click here for file
