# Supplementary material for: Bezafibrate improves postprandial hypertriglyceridemia and associated endothelial dysfunction in patients with metabolic syndrome: a randomized crossover study
Source: Cardiovasc Diabetol. 2014 Apr 5;13:71. doi: 10.1186/1475-2840-13-71 (PMC4108061; doi:10.1186/1475-2840-13-71)
Supplement: Additional file 1: Table S1 — Postprandial changes in lipid profiles and glycemic parameters in the bezafibrate and control groups. [file 1475-2840-13-71-S1.doc]

**Table S1. Postprandial changes in lipid profiles and glycemic parameters in the bezafibrate and control groups**

|  | **Fasting** | **2 h** | **4 h** | **6 h** | **8 h** | **Total AUC** |
| --- | --- | --- | --- | --- | --- | --- |
| **Lipid profiles** |  |  |  |  |  |  |
| Total-C (mg/dl)  Bezafibrate  Control | 197 ± 8  217 ± 9 | 197 ± 9  218 ± 9 | 197 ± 9  217 ± 9 | 200 ± 9  2220 ± 10 | 196 ± 9  223 ± 9 | 1580 ± 72  1749 ± 76* |
| LDL-C (mg/dl)  Bezafibrate  Control | 129 ± 6  135 ± 9 | 126 ± 6  133 ± 10 | 122 ± 5  128 ± 10 | 125 ± 5  132 ± 10 | 127 ± 5  134 ± 9 | 1005 ± 42  1058 ± 77 |
| HDL-C (mg/dl)  Bezafibrate  Control | 46 ± 2  43 ± 2 | 45 ± 2  41 ± 2 | 43 ± 2  39 ± 1 | 45 ± 2  40 ± 1 | 45 ± 2  42 ± 2 | 356 ± 19  328 ± 11 |
| TG (mg/dl)  Bezafibrate  Control | 135 ± 24*  203 ± 35 | 202 ± 33*  300 ± 41 | 247 ± 36*  402 ± 55 | 209 ± 27*  383 ± 58 | 160 ± 29*  319 ± 54 | 1614 ± 240*  2694 ± 342 |
| RLP-C (mg/dl)  Bezafibrate  Control | 5.7 ± 0.7  7.9 ± 1.3 | 8.5 ± 1.2  12.2 ± 1.7 | 11.8 ± 1.5  19.6 ± 3.0 | 9.5 ± 1.1  19.7 ± 3.6 | 7.7 ± 1.2  17.8 ± 3.8 | 73.2 ± 9.0*  128.7 ± 14.1 |
| ApoB-48 (μg/ml)  Bezafibrate  Control | 3.5 ± 0.7*  6.0 ± 1.5 | 7.4 ± 0.9*  9.3 ± 1.5 | 8.7 ± 1.6*  11.7 ± 2.0 | 6.9 ± 1.2*  11.7 ± 2.3 | 5.3 ± 1.1*  9.6 ± 2.2 | 55.2 ± 9.3*  81.4 ± 13.5 |
| Insulin (μU/ml)  Bezafibrate  Control | 7.8 ± 1.6  8.5 ± 1.4 | 82.1 ± 23.6  59.1 ± 13.3 | 47.8 ± 9.2  38.9 ± 7.3 | 32.3 ± 10.2  24.5 ± 5.8 | 14.4 ± 3.6  12.0 ± 3.2 | 343.5 ± 83.0  265.8 ± 51.3 |
| Glucose (mg/dl)  Bezafibrate  Control | 97 ± 3  101 ± 5 | 133 ± 15  128 ± 13 | 117 ± 12  108 ± 11 | 94 ± 8  112 ± 14 | 93 ± 19  100 ± 10 | 881 ± 75  898 ± 89 |
| Pentraxin 3 (ng/ml)  Bezafibrate  Control | 1.5 ± 0.2  1.5 ± 0.2 | 1.5 ± 0.2  1.4 ± 0.1 | 1.3 ± 0.1  1.4± 0.1 | 1.2 ± 0.1  1.3 ± 0.2 | 1.2 ± 0.1  1.3 ± 0.1 | 11.0 ± 1.0  11.1 ± 0.9 |
| **Endothelial function** |  |  |  |  |  |  |
| Brachial artery diameter (mm)  Bezafibrate  Control | 4.3 ± 0.2  4.3 ± 0.2 | 4.3 ± 0.1  4.4 ± 0.2 | 4.4 ± 0.1  4.5 ± 0.2 | 4.4 ± 0.1  4.4 ± 0.2 | 4.4 ± 0.2  4.4 ± 0.2 |  |
| Maximum increase in flow (%)  Bezafibrate  Control | 432 ± 50  435 ± 54 | 435 ± 79  442 ± 54 | 438 ± 49  446 ± 43 | 441 ± 46  440 ± 57 | 440 ± 60  440 ± 64 |  |
| %FMD  Bezafibrate  Control | 6.9 ± 0.7*  5.9 ± 0.9 | 5.4 ± 0.7*  4.8 ± 0.8 | 4.9 ± 0.8*  3.6 ± 0.7 | 5.9 ± 0.6*  5.3 ± 0.6 | 7.5 ± 0.7*  6.3 ± 0.8 |  |

Values are the mean ± SE. Total-C, total cholesterol; LDL-C, low-density lipoprotein cholesterol; HDL-C, high-density lipoprotein cholesterol; TG, triglyceride; RLP-C, remnant lipoprotein cholesterol; ApoB-48, apolipoprotein B-48; FMD, flow-mediated dilation; AUC, area under the curve. *p < 0.05, vs. control group after treatment for 4 weeks.
