# Supplementary material for: Bezafibrate improves postprandial hypertriglyceridemia and associated endothelial dysfunction in patients with metabolic syndrome: a randomized crossover study
Source: Cardiovasc Diabetol. 2014 Apr 5;13:71. doi: 10.1186/1475-2840-13-71 (PMC4108061; doi:10.1186/1475-2840-13-71)
Supplement: Additional file 2: Table S2 — TG and cholesterol concentrations in lipoprotein fractions in the chylomicron, VLDL, LDL, and HDL fractions. [file 1475-2840-13-71-S2.doc]

**Table S2.**

**Triglyceride and cholesterol concentrations in lipoprotein fractions in the chylomicron, VLDL, LDL and HDL size ranges.**

|  | **Chylomicron** | **VLDL** | **LDL** | **HDL** |
| --- | --- | --- | --- | --- |
| **Triglyceride (mg/dl)** |  |  |  |  |
| Fasting state  Bezafibrate  Control | 7 ± 2  11 ± 2 | 70 ± 16*  106 ± 26 | 38 ± 4*  52 ± 5 | 27 ± 4*  33 ± 4 |
| 4 h after cookie ingestion  Bezafibrate  Control | 13 ± 3*  24 ± 3 | 152 ± 30*  265 ± 51 | 54 ± 4*  65 ± 4 | 30 ± 5*  40 ± 6 |
| **Cholesterol (mg/dl)** |  |  |  |  |
| Fasting state  Bezafibrate  Control | 3.4 ± 0.2  4.4 ± 0.6 | 33.5 ± 2.5*  38.0 ± 2.8 | 124.6 ± 11.3*  131.5 ± 7.8 | 43.1 ± 2.8  43.6 ± 2.1 |
| 4 h after cookie ingestion Bezafibrate  Control | 4.0 ± 0.3*  6.5 ± 0.7 | 34.6 ± 2.5*  41.4 ± 2.7 | 124.9 ± 6.5*  131.1 ± 8.0 | 42.0 ± 2.7  41.45 ± 1.9 |

Values are the mean ± SE. VLDL, very low-density lipoprotein; LDL, low-density lipoprotein; HDL, high-density lipoprotein; *p < 0.05, vs. control group.
